# VIP Deficient Mice Exhibit Resistance to Lipopolysaccharide Induced Endotoxemia with an Intrinsic Defect in Proinflammatory Cellular Responses

**DOI:** 10.1371/journal.pone.0036922

**Published:** 2012-05-17

**Authors:** Catalina Abad, Yossan-Var Tan, Gardenia Cheung-Lau, Hiroko Nobuta, James A. Waschek

**Affiliations:** 1 Department of Psychiatry, David Geffen School of Medicine, University of California Los Angeles, Los Angeles, California, United States of America; 2 Department of Pathology and Lab Medicine, David Geffen School of Medicine, University of California Los Angeles, Los Angeles, California, United States of America; 3 Department of Biological Chemistry, David Geffen School of Medicine, University of California Los Angeles, Los Angeles, California, United States of America; Charité-University Medicine Berlin, Germany

## Abstract

Vasoactive intestinal peptide (VIP) is a pleiotropic neuropeptide with immunomodulatory properties. The administration of this peptide has been shown to have beneficial effects in murine models of inflammatory diseases including septic shock, rheumatoid arthritis, multiple sclerosis (MS) and Crohn's disease. However, the role of the endogenous peptide in inflammatory disease remains obscure because VIP-deficient mice were recently found to exhibit profound resistance in a model of MS. In the present study, we analyzed the response of female VIP deficient (KO) mice to intraperitoneal lipopolysaccharide (LPS) administration. We observed significant resistance to LPS in VIP KO mice, as evidenced by lower mortality and reduced tissue damage. The increased survival was associated with decreased levels of proinflammatory cytokines (TNFα, IL-6 and IL-12) in sera and peritoneal suspensions of these mice. Moreover, the expression of TNFα and IL-6 mRNA was reduced in peritoneal cells, spleens and lungs from LPS-treated VIP KO vs. WT mice, suggesting that the resistance might be mediated by an intrinsic defect in the responsiveness of immune cells to endotoxin. In agreement with this hypothesis, peritoneal cells isolated from VIP KO naive mice produced lower levels of proinflammatory cytokines in response to LPS *in vitro*. Finally, decreased NF-κB pathway activity in peritoneal cells was observed both *in vivo* and *in vitro*, as determined by assay of phosphorylated I-κB. The results demonstrate that female VIP KO mice exhibit resistance to LPS-induced shock, explainable in part by the presence of an intrinsic defect in the responsiveness of inflammatory cells to endotoxin.

## Introduction

Inflammation is an essential mechanism of self-protection against pathogen invasion and injury, and involves innate and adaptive immune mechanisms [1]. Macrophages, critical effector cells of the innate immune response, play a major role in this process by releasing pro-inflammatory cytokines such as tumor necrosis factor (TNF)-α, interleukin (IL)-1β, IL-6 and IL-12, and chemokines such as monocyte chemotactic protein (MCP)-1 or macrophage inflammatory protein (MIP)-1α that promote leukocyte recruitment to the damaged tissues [2]. In turn, the main cellular effectors of innate immunity, macrophages and neutrophils, engulf and digest bacteria, remove cell debris, and produce growth factors to facilitate the healing process [2], [3], [4]. However, the ability of the immune system to mediate direct or indirect killing of cells and pathogens make it a potential threat to host survival. As a consequence, autoimmunity and acute and chronic inflammatory diseases are triggered by immune dysfunction. In this regard, septic shock is a serious acute inflammatory condition often caused by an overwhelming infection that usually leads to impaired perfusion and multiple organ failure [5]. The most frequent microbial agents responsible for septicemia are Gram-negative bacteria. In addition to the release of bacterial toxins, lipopolysaccharide (LPS), a major component of Gram negative bacteria outer membranes, binds preferentially to toll-like receptor (TLR)-4 expressed by many immune and non-immune cell types [6]. This triggers the activation of the nuclear factor kappa B (NF-κB) pathway, which causes the release of inflammatory mediators [7], [8]. Pathology arises when the presence of excessive levels of endotoxin due to bacterial overgrowth leads to a hyperactivation of macrophages and granulocytes that massively invade all tissues and release proinflammatory mediators. This phenomenon can be experimentally mimicked in animals by administration of an exogenous high dose of LPS.

Although proper control of the immune response is not always achievable, the organism has developed different endogenous mechanisms to control the inflammatory response and preserve homeostasis [9]. These include the production of soluble mediators such as the anti-inflammatory cytokine IL-10, heat shock proteins, certain prostaglandins, and hormones such as cortisol. Lately, several neuropeptides have been suggested to be immune modulators that dampen the inflammatory response, including the structurally-related peptides VIP and PACAP (vasoactive intestinal peptide and pituitary adenylate cyclase-activating polypeptide, respectively) [10]. These natural inhibitors of immunity are currently being tested as potential therapeutic candidates for the treatment of inflammatory diseases.

VIP is a versatile 28-amino acid neuropeptide with thoroughly described anti-inflammatory functions [11], [12]. VIP binds mainly to two receptors, named VPAC1 and VPAC2, which belong to the G-coupled protein receptor family and are expressed on many different immune cell types [13]. Numerous *in vitro* and *in vivo* studies have demonstrated that VIP, *via* actions on these receptors, is able to suppress the production and/or release of key molecules for the inflammatory response, such as proinflammatory cytokines, and chemokines [14], [15]. In fact, the potential use of VIP as a treatment for acute and chronic inflammatory diseases including septic shock, rheumatoid arthritis, Crohn's disease and multiple sclerosis has been suggested based on positive clinical and pathological outcomes in corresponding murine models of inflammatory diseases [16], [17], [18], [19]. In addition, it has been demonstrated that the systemic endogenous levels of this neuropeptide increase in response to an inflammatory challenge like endotoxic shock in humans or LPS-induced endotoxemia in mice [20], [21]. As VIP has been reported to be produced by neurons, endocrine and immune cells, multiple tissular and cellular sources can contribute to the increase of VIP after inflammation. For example, it has been recently shown that LPS induces the production of VIP by chromaffin cells from the adrenal medulla [22]. In addition, VIP levels are upregulated in cell suspensions from primary and secondary lymphoid organs in response to LPS treatment [23]. Whether or not this upregulation has an impact on the course of the inflammatory response is largely unknown. VIP-deficient mice (KO) display certain physiological abnormalities such as disrupted circadian rhythms, airway hyperresponsiveness to the cholinergic agonist methacholine, and pulmonary hypertension [24], [25], [26]. In addition, we have recently reported that despite the well-described anti-inflammatory actions of VIP, VIP KO female mice were unexpectedly resistant to experimental autoimmune encephalomyelitis (EAE) induction, with reduced immune cell infiltration of the spinal cord and brain parenchyma [27]. Potential mechanisms for this resistance may include defects in the innate arm of immunity. As a model to test this hypothesis, we investigated the response of VIP KO mice to LPS-induced endotoxemia. We found that female VIP KO mice exhibited reduced mortality in response to LPS. This phenotype was associated with reduced inflammatory damage in the lungs of the KO mice, and reduced levels of proinflammatory mediators in the sera and peritoneal suspensions. Moreover, we found reduced mRNA expression of TNFα and IL-6 in peritoneal cells, spleens and lungs of LPS-treated VIP KO mice. Interestingly, peritoneal cells isolated from naive VIP KO mice produced lower levels of proinflammatory cytokines than wild type [2] cells in response to LPS. Finally, NF-κB activation by LPS was reduced in cells from VIP KO mice.

## Materials and Methods

### Mice

All animal studies were approved by the institutional animal research committee of the University of California at Los Angeles (UCLA) Female 6- to 8-week-old mice WT and VIP KO mice on a C57BL/6 background (backcrossed for at least twelve generations) [25] were maintained under specific pathogen-free conditions. Experimental procedures followed the recommendations for animal use and welfare, as dictated by the UCLA Division of Laboratory Animals and the guidelines from the National Institutes of Health.

### Induction of endotoxemia and histology

Endotoxemia was induced in mice by intraperitoneal (i.p.) injection of 40 mg/Kg of LPS (*Salmonella enteritidis*; Sigma, St. Louis, MO), and survival was monitored thereafter for at least one week. For histopathological studies, separate cohorts of mice were sacrificed 24 hours later and lungs were removed and fixed in Bouin's solution for 2 hours. Fixed tissues were then dehydrated and embedded in paraffin, sectioned, and stained with hematoxylin and eosin for morphological examination. The degree of inflammation was scored from 0 to 3 by two independent researchers in a blinded fashion as described [28]: 0 - all alveolar septae thin and delicate, no hemorrhage or fibrosis present; 1 - congested alveolar septae in less than 1/3 of the field, mild hemorrhage and fibrosis in less than 1/3 of the field; 2- congested alveolar septae 1/3 to 2/3 of the field, moderate hemorrhage and fibrosis in 1/3 to 2/3 of the field, 3: congested alveolar septae in more than 2/3 of the field, severe hemorrhage and fibrosis in more than 2/3 of the field.

### Collection of sera and peritoneal supernatants

For cytokine measurements, mice were sacrificed 3 and 6 hours after LPS injection (or 3, 6 and 24 hours for IL-10), blood was collected by intracardiac puncture under anesthesia, and serum was obtained after clotting for 2 hours at room temperature. In order to collect peritoneal suspensions at these time points, mice were injected i.p. with 2 ml of RPMI 1640 (Gibco, Gaithersburg, MD) and fluid was collected after abdominal massage. In both cases, samples were centrifuged for 10 min at 2000 rpm and supernatants were stored at −20°C until assay.

### RNA isolation and Real time-PCR analysis

RNA was extracted from cells from peritoneal suspension collected as above, and from spleens and lungs at the indicated time points with Trizol (Invitrogen, Carlsbad, CA) as recommended by the manufacturer. RNA was resuspended in diethylpyrocarbonate (DEPC) water and quantified at 260/280 nm. cDNA from 1 µg of RNA was synthesized with the iScriptTM cDNA synthesis Kit from Bio-Rad (Hercules, CA). Quantitative real-time PCR analysis was performed using the iQ™ SYBR Green Supermix from Bio-Rad in triplicate in 25 µl reaction volumes. The sequences of the primers used were: mouse HPRT 5′-TGGTGAAAAGGACCTCTCGAA-OH sense and 5′-TCAAGGGCATATCCAACAACA-OH antisense, mouse TNFα sense 5′-CGATCACCCCGAAGTTCAGTA-OH and 5′-GGTGCCTATGTCTCAGCCTCTT-OH antisense, mouse IL-6 5′-TTCCATCCAGTTGCCTTCTTG–OH sense and 5′-TTGGGAGTGGTATCCTCTGTGA–OH antisense. The GenBank accession numbers for the PCR products are: HPRT, NM013556; TNFα, NM013693; IL-6, NM031168. The amplification conditions were 4 min at 95°C followed by 40 cycles of denaturation at 96°C for 20 sec, annealing at (60°C for IL-6, 62°C for TNFα), for 30 sec and extension at 72°C for 30 sec. The specificity of gene amplification was confirmed by sequencing of the PCR products and melting curves analysis. Comparison of specific ratios (gene of interest/house keeping gene) was used to assess differences between groups.

### *In vitro* studies

For cell culture studies, peritoneal lavage was obtained as described above. Whole peritoneal cells were washed and cultured in 96 well tissue culture plates at 2×10^5^ cells/well in 200 µl/well with RPMI 1640 complete medium (2% FCS, 2 mM L-glutamine, 100 U/ml penicillin, and 100 µg/ml streptomycin) and 10 ng/ml of LPS (*Salmonella enteritidis*; Sigma) at 37°C in a 5% CO_2_ humidified atmosphere. Supernatants were collected at different time points, centrifuged at 2000 rpm and stored at −20°C until assay of cytokines by ELISA.

### ELISA

TNFα, IL-6, IL-10 and IL-12p40 quantities in serum, peritoneal suspension or culture supernatants were determined by standard sandwich ELISA. Development kits were used according to manufacturer instructions (Peprotech, Rocky Hill, NJ). Briefly, 96 well plates (Corning, NY) were coated with capture antibody O/N at room temperature. After blocking for 1 hour with PBS/1%BSA, samples and standards were added O/N at 4°C. Plates were then incubated with detection antibody for 2 hours, and avidin/peroxidase was added for 30 min. Washing four times with PBS/0.05%Tween was performed before the addition of all reagents. Finally, plates were incubated with substrate solution and A_405 nm_ was measured 30 minutes later with a Bio-Rad microplate reader.

### Western blot

For analysis of freshly isolated peritoneal cells (*in vivo*), mice were injected with LPS and cells were collected one hour later from peritoneal lavage. For *in vitro* studies, peritoneal cells were incubated with 10 ng/ml of LPS as above and collected 15 min later. In both cases cells were centrifuged and pellets were frozen in dry ice. Pelletted cells were homogenized in RIPA buffer containing protease inhibitor cocktail (Roche, Indianapolis, IN) and phosphatase inhibitor cocktail (Sigma). Concentration of total protein was determined using a BCA kit (GE healthcare, Piscataway, NJ) and 20 µg of protein was resolved on a 4–20% gradient polyacrylamide gel (Invitrogen) and transferred to PVDF membranes (Bio-Rad). Membranes were then incubated with primary antibody for phosphorylated IκB (Santa Cruz, CA), appropriate secondary antibody conjugated to horse-radish peroxidase (Cell Signaling, Danvers, MA), and visualized using ECL plus (GE healthcare). During the steps of blocking, primary and secondary antibody incubations for p-IκB, the same phosphatase inhibitor cocktail was added. Films were scanned, and band density was quantified using digital image densitometry analysis (ImageJ; National Institutes of Health, Bethesda, MD).

### Statistical analysis

Graphs were created with the GraphPad 4.0 Prism software. All results were expressed as means ± SEM. Survival curves were analyzed by Logrank (Mantel-Haenszel) test and ELISA, Real time-PCR and Western blot data were analyzed by ANOVA and Student *t*-test, with p<0.05 as the minimum level of significance.

## Results

### Increased survival of VIP KO vs WT mice after LPS administration

**Figure 1:**
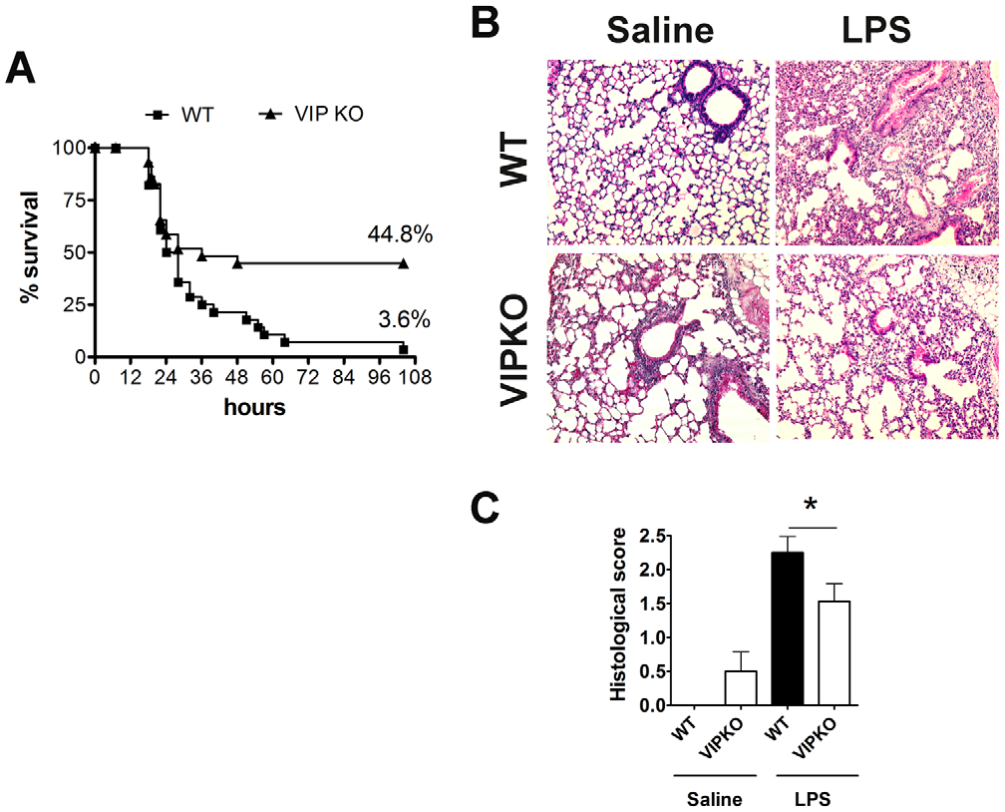
VIP KO mice exhibit reduced mortality and lung histopathology in response to LPS injection. Female WT (C57BL6) and VIP KO mice were injected i.p. with LPS (40 mg/Kg). A, Kaplan Meier curve analysis of survival cumulative of four experiments (total WT n = 29; VIP KO n = 28) (Curve comparison Logrank test **p<0.01). B, Representative sections of lungs from control (noninjected) or LPS-injected WT and VIP KO mice (24 hours post injection) stained with H&E. C, Histological scores of LPS-injected WT vs. VIP KO mice (mean of two experiments; total WT n = 7; VIP KO n = 9), 24 hours after LPS injection, scored from 0 to 3 according to the level of lung inflammation as described in *Materials and Methods*. (Student's *t*-test *p<0.05).

We previously reported that female VIP KO mice exhibited a paradoxical resistance to EAE induction [27]. To determine if the resistance in these mice could potentially be explained by a defect in innate immunity, we injected LPS (40 mg/kg) i.p. to female WT and VIP KO mice and monitored their survival over time. The administration of this endotoxin triggers an acute inflammatory response leading to multiple organ dysfunction and subsequently death in wild type mice within two to three days. Figure 1A depicts the Kaplan-Meier analysis of WT vs. VIP KO survival of four experiments. In the first 24 hours after LPS injection, WT and VIP KO mice succumbed to LPS at a similar rate, with 50.0% and 58.6% survival for WT and VIP KO mice, respectively, and no significant differences between the two curves. After this time point, the mortality rates dramatically diverged, with few deaths occurring in the VIP KO group in the next 24 hours, and none after 48 hours post-injection. At the end of the study (5 days after LPS administration), a significantly higher overall survival of VIP KO mice (44.8%) vs WT mice (3.6%) was observed (**p = 0.0049; curve comparison Logrank test). For histopathological studies we chose the lung, a main target tissue for immune cell infiltration and inflammation in this model of LPS-induced endotoxemia. After LPS administration, lung inflammatory injury is characterized by patchy areas of neutrophilic infiltrates with thickening of the alveolar septae. Interestingly, we found a mild degree of basal inflammation in female VIP KO mice lungs (0.5±0.29 histological score) compared to those of WT controls (Figure 1B), as it has been previously reported in males [24]. However, in agreement with our clinical data, whereas the lungs of WT mice exhibited severe infiltration by immune cells 24 hours after LPS, those of the VIP KO mice were significantly less inflamed and exhibited a better preserved architecture (Figure 1B). Indeed, the average histopathological score post-LPS administration was 2.25±0.24 for WT mice, vs 1.53±0.26 for VIP KO mice (*p<0.05) (Figure 1C). Interestingly, we did not observe such a resistant clinical phenotype to LPS in VIP KO male mice (data not shown), suggesting that the effects of chronic absence of VIP could be sex dependent. In this sense, sex differences in the composition of the peritoneal cell population have been reported [29].

### Proinflammatory cytokine levels are reduced in sera and peritoneal suspensions of VIP KO mice after LPS administration

**Figure 2:**
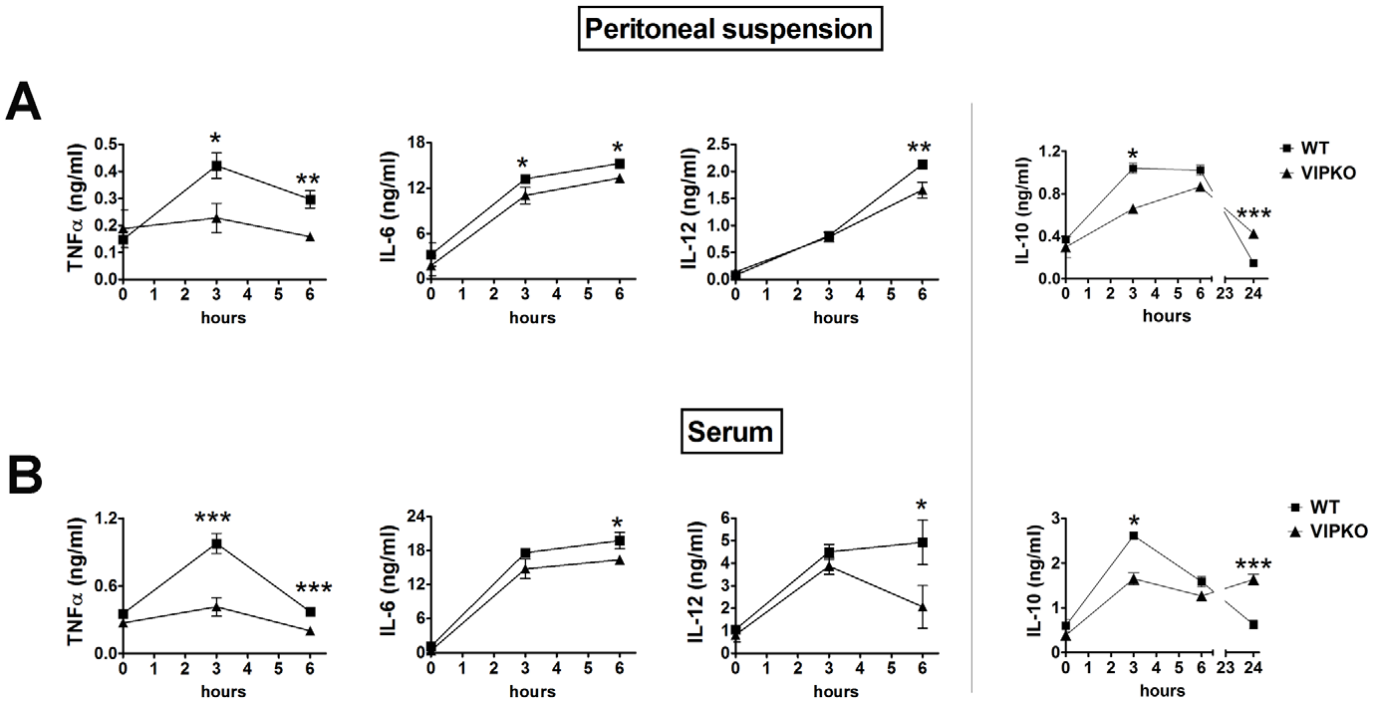
VIP KO mice exhibit reduced levels of proinflammatory cytokines in the peritoneal fluid and serum. Female WT (C57BL6) (n = 6) and VIP KO mice (n = 6) were injected i.p. with LPS (40 mg/Kg), and serum and peritoneal suspensions were collected 0, 3 and 6 (and also 24 for IL-10) hours post-injection. The levels of TNFα, IL-6, IL-12p40 and IL-10 were assessed by sandwich ELISA as described in *Materials and Methods*. Student's *t*-test *p<0.05; **p<0.01. One of three representative experiments is shown.

LPS triggers the secretion of proinflammatory cytokines by a wide array of cell types as a part of the innate immune response. In order to determine if the ability of VIP KO mice to develop such a response was impaired, we measured the protein levels of TNFα, IL-6 and IL12p40 in the sera and the peritoneal suspensions of LPS injected mice by ELISA. As shown in Figure 2, the levels of all these cytokines increased in the serum and peritoneal fluid of the WT mice in response to LPS as expected, with different time-courses. For TNFα, there was an early induction with a peak only 3 hours after the injection of LPS in both types of samples (3-fold increase compared to basal levels). For IL-6 and IL-12, the levels increased progressively reaching the maximum 6 hours after the induction of the disease (3-fold or higher fold increase over controls). Supporting the clinical phenotype of VIP KO mice, even though we found an increase of proinflammatory cytokines after LPS injection in the sera and peritoneal suspensions of these mice, the levels of these cytokines were lower than those in the WT mice. The reduction in cytokine levels was particularly striking for TNFα 3 hours post-injection (0.42±0.05 ng/ml in WT vs 0.23±0.05 ng/ml in VIP KO mice; *p<0.05 in peritoneal suspensions and 0.98±0.09 ng/ml in WT vs 0.41±0.08 ng/ml in VIP KO mice; ***p<0.001 in sera). In addition, we measured the levels of the antiinflammatory cytokine IL-10. As for the proinflammatory cytokines analyzed, the levels of IL-10 were lower in VIP KO mice than in WT mice at early time points, especially 3 hours after LPS administration. Because IL-10 plays a role in the resolution phase of inflammation, we also measured its levels at a later time point, 24 hours post-LPS injection, which is the time point after which the death rate decreased in the VIP KO group. Interestingly, the levels of IL-10 in both peritoneal suspensions and sera were significantly higher in VIP KO mice at this time (***p<0.001). The findings that the KO mice produced lower amounts of TNFα, IL-6, IL-12p40 and IL-10 at early time points and produced more IL-10 at later time points suggest a depressed response of the innate arm of immunity, and correlate with the decreased mortality in the VIP KO mice.

### VIP KO mice express lower levels of proinflammatory mediators in peritoneal cells, spleen and lung

**Figure 3:**
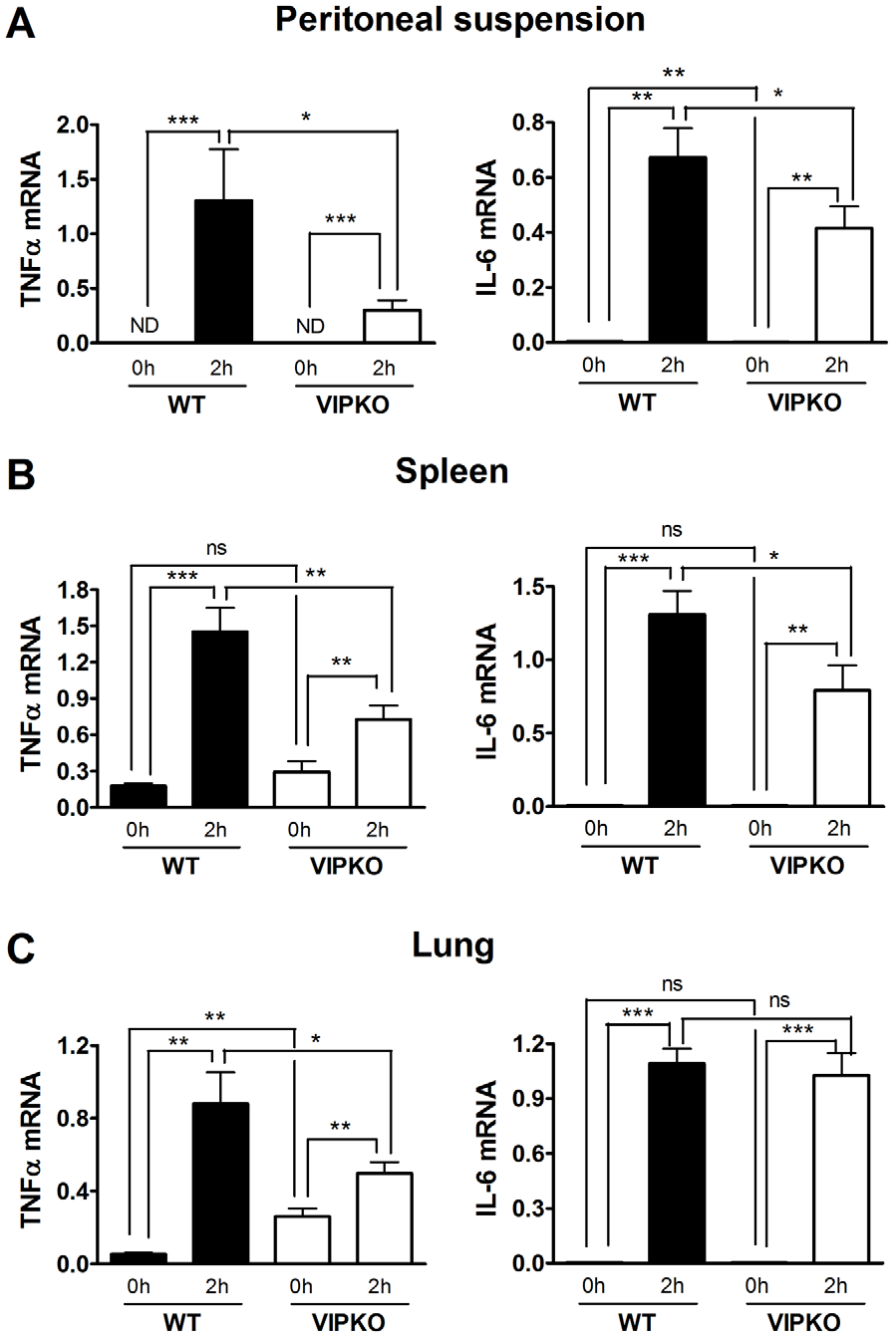
Levels of TNFα and IL-6 gene expression in peritoneal cells, spleen and lung after LPS injection is reduced in VIP KO vs. WT mice. Female WT (C57BL6) (n = 6) and VIP KO mice (n = 6) were injected i.p. with LPS (40 mg/Kg), and cells or tissues were collected 0 and 2 hours post-injection and stored at −80°C until further analysis. RNA was extracted with the Trizol method, and the mRNA expressions of TNFα and IL-6 were assessed by Real time RT-PCR. The mean values ± SEM are shown (n = 6/group). Student's *t*-test *p<0.05.

Peritoneal cells are the first immune cell population exposed to LPS in this model. However, several studies have shown that LPS rapidly access the circulation after intraperitoneal administration and that its levels do not drop as quickly despite the clearing activity of liver cells [30]. Therefore, circulating LPS triggers a global inflammatory response whereby cells in different organs such as the spleen and lung become activated and produce proinflammatory mediators. These in turn promote immune cell migration and tissue infiltration. Immigrant immune cells can in turn amplify inflammation by producing more proinflammatory cytokines. As shown above, the systemic levels of proinflammatory cytokines were reduced in the VIP KO mice. In order to investigate the source of this deficiency, we measured by real time RT-PCR the mRNA levels of IL-6 and TNFα as representative molecules of the inflammatory cascade, in peritoneal cells, spleen and lung two hours after LPS injection. As expected (Figure 3A–C), the mRNA levels of these two proinflammatory markers were increased in all the WT mice tissues at 2 hours post-LPS injection. However, the induction of these cytokines in VIP KO mice was much less pronounced than in WT mice in all organs analyzed. Only gene expression of IL-6 in the lung after LPS was not different between WT and VIP KO mice. Interestingly, the mRNA levels of TNFα in non-injected VIP KO lungs were higher than in the WT mice, in agreement with the mild basal inflammation found at the histological level in this organ in the KO mice (**p<0.01). Our results suggest that in the chronic absence of VIP, the LPS-induction of proinflammatory mediators was impaired.

### Peritoneal cells from VIP KO mice exhibit a reduced cytokine response to LPS *in vitro*

**Figure 4:**
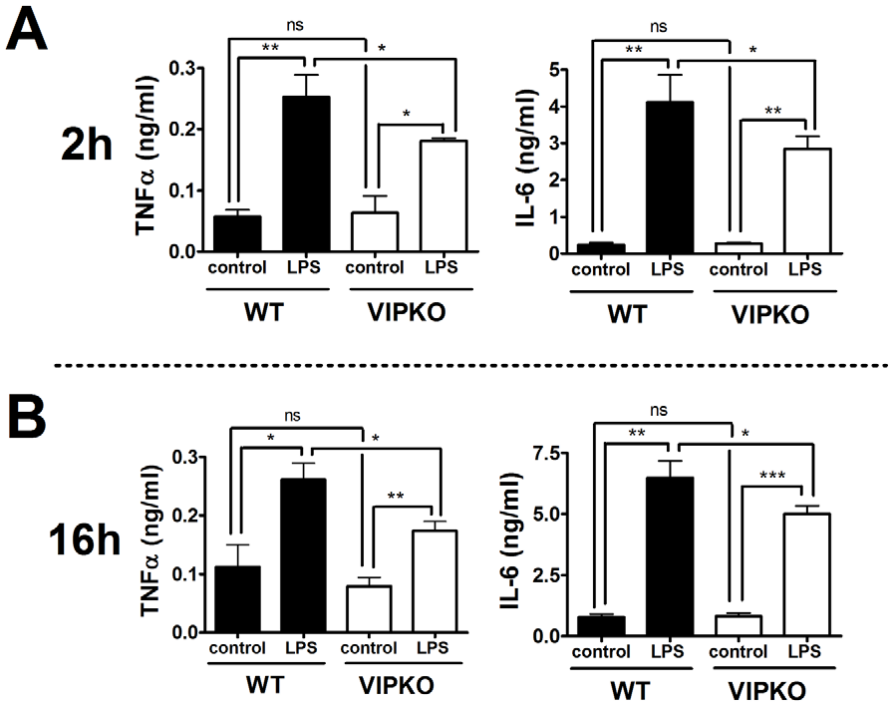
Peritoneal cells from VIP KO mice exhibit an intrinsic defect in cytokine response to LPS- administration. Peritoneal cells were collected from WT (n = 3) and VIP KO mice (n = 3), and cultured in complete RPMI in triplicate in the presence or absence of LPS (10 ng/ml). Supernatants were collected 2 (A) and 16 h (B) later, and stored at −20°C for analysis of TNFα and IL-6 levels by ELISA. Student's *t*-test *p<0.05; **p<0.01; ***p<0.001. Representative data are shown of four independent experiments.

In order to test whether the attenuated response to LPS in the VIP KO mice was due to an intrinsic defect in the ability of immune cells to mount an inflammatory response, we isolated and cultured whole peritoneal cells from WT and VIP KO mice in the presence or absence of LPS. Supernatants were collected to measure the levels of TNFα and IL-6 by ELISA (Figure 4A, B). We found that 2 hours after LPS stimulation, WT cells secreted TNFα and IL-6 as expected (Figure 4A). In comparison, VIP KO cells released lower cytokine levels (*p<0.05 for both cytokines). To assess the possibility that cytokine release may be delayed in VIP KO cells, we also collected the supernatants of similar cultures 16 hours after LPS stimulation (Figure 4B). Again, VIP KO cells produced significantly lower amounts of TNFα and IL-6 at this time point (*p<0.05). These *in vitro* experiments suggest that VIP KO mice may present a defect in immune cells to respond to LPS.

### NF-κB signaling is diminished in VIP KO peritoneal cells *in vivo* and *in vitro* after LPS administration

**Figure 5:**
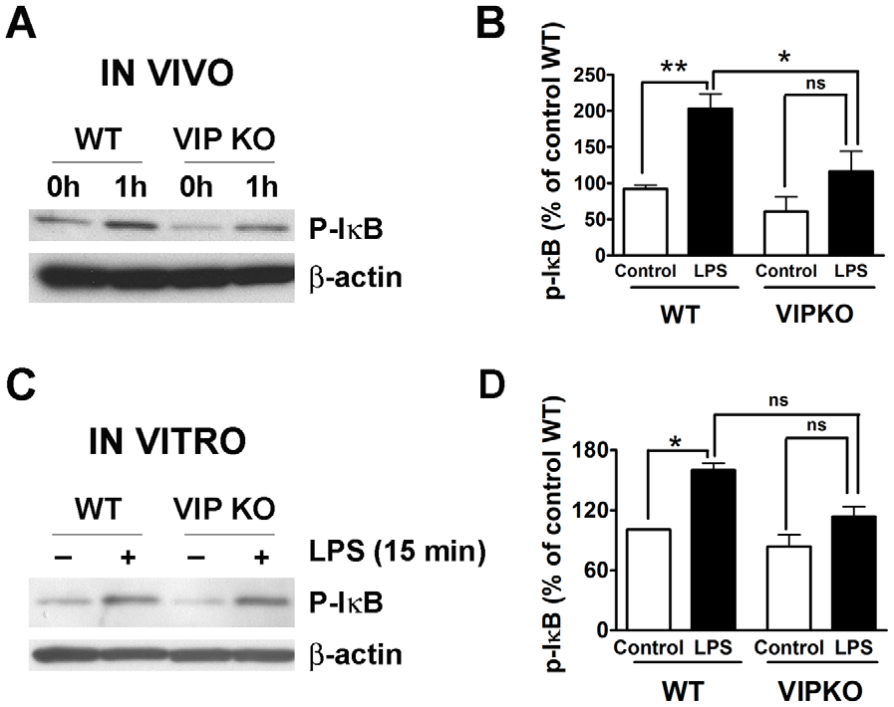
LPS-induced activation of the NF-κB pathway is diminished in VIP KO mice. Activation of the NF-κB signaling pathway was at several time point determined by measurement of p-IκB protein levels by Western blot in (A, B) peritoneal cells isolated from WT and VIP KO mice without or 1 hour after injection of LPS (40 mg/Kg), and (C, D) peritoneal cells isolated from WT and VIP KO mice and stimulated in culture with or without LPS (10 ng/ml). Bar graphs represent the quantification of band density. Student's *t*-test *p<0.05, ns = not significant. A representative blot of three experiments is shown.

The inflammatory cascade usually proceeds with the activation of the NF-κB pathway, which leads to the synthesis of chemokines and cytokines which amplify the immune response. In order to identify potential defects leading to the resistance of VIP KO mice to LPS, we studied the activity of this pathway *in vitro* and *in vivo* by Western blot (Figure 5A, B). As a readout of NF-κB activation, we studied the amount of phosphorylated-IκB (p-IκB) in protein extracts from whole peritoneal cells isolated from WT and VIP KO mice in basal conditions and 1 hour after LPS injection (Figure 5A). Interestingly, the levels of p-IκB were already lower in non-injected VIP KO mice compared to WT controls. The levels of p-IκB increased in both WT and VIP KO mice post-LPS injection. However, the levels of this signaling mediator after LPS were significantly lower in VIP-deficient mice, suggesting a reduced responsiveness of the NF-κB pathway in these mice. In addition, we measured p-IκB in peritoneal cells cultured with and without LPS as we did above for cytokine measurements (Figure 5B). Similar to the results found *in vivo*, whereas LPS treatment significantly elevated the levels of p-IκB in peritoneal cells from WT mice (*p<0.05), it only triggered a modest increase of p-IκB in cells from VIP KO mice (p = 0.15). Indeed, the levels of p-IκB in cells treated with LPS where lower in VIP KO mice than in WT mice, although the difference was not significant (p = 0.08). The reduction of NF-κB signaling correlates with the impaired secretion of proinflammatory cytokines in the VIP KO mice.

## Discussion

The antiinflammatory actions of VIP have been repeatedly demonstrated both *in vitro* and *in vivo*, suggesting the therapeutic potential of this peptide [11]. However, using the MOG_35–55_ immunization model, we recently reported that female VIP KO mice exhibited an unexpected resistance to EAE induction [27], suggesting that the activities of endogenous VIP in the immune system may be complex. During the pathogenesis of EAE, both the innate and adaptive arms of the immune system are involved [31]. Interestingly, we did not find a defect in the lymphocyte priming phase in the VIP KO mice in our prior EAE study, because T cells from immunized VIP KO mice showed robust responses to MOG *in vitro* on antigen rechallenge experiments [27]. In addition, it has been recently shown that VIP KO mice exhibited less weight loss and improved survival to murine CMV infection, a phenotype which was associated with enhanced adaptive antiviral cellular immunity [32]. Thus, the activation of CD4 and CD8 T cells following immunization does not appear to be impaired in these mice. On the other hand, adoptive transfer of T cells from MOG_35–55_-immunized WT mice induced EAE in WT but not VIP KO mice [27], suggesting that impairments in one or more cell types other than T cells might explain the EAE resistance in the latter mice. Here, we used the LPS model of endotoxemia and we found that VIP KO mice exhibited reduced mortality, less tissue injury, and impaired proinflammatory responses. Because the innate immune system is the major component in the pathogenesis of this model, these results suggest that these mice may exhibit a defect in this arm of immunity. We investigated potential deficiencies in TNFα and IL-6 in these mice, as these are two cytokines critically involved in the acute inflammatory cascade. In this sense, TNFα is rapidly produced by immune cells and is a major activator of the NF-κB pathway, which triggers the expression of chemokines and other proinflammatory cytokines, amplifying the immune response [33], IL-6, in addition to generally being an inflammatory cytokine, activates the coagulatory cascade, and if present in excessively high levels, can lead to disseminated intravascular coagulation, impaired reperfusion and death [34]. In our study, we found a reduction in the systemic levels of both TNFα and IL-6 in VIP KO mice injected with LPS. Although multiple cell types can produce these cytokines, our analysis of peritoneal cells infers that VIP KO mice may exhibit a defect in immune cells, most likely within the myeloid component, because the pool of peritoneal cells is highly represented by myeloid cells including monocytes, macrophages and granulocytic populations, and these cells are the main contributors to the rapid increase in TNFα and IL-6 levels in response to LPS. It has been shown that peritoneal cells play an important role in LPS-induced endotoxemia. First of all, this is the initial immune cell population exposed to the intraperitoneal LPS injection, and all cell components express TLRs. Moreover, it has been shown that peritoneal lavage performed before LPS administration to remove these cells reduces the elevation of serum TNFα and IL-6 and mortality in mice [35]. Here, we found decreased TNFα and IL-6 mRNA levels in peritoneal cells from VIP KO mice injected with LPS, and that these cells responded poorly to LPS *in vitro*. This reduction suggests that the intrinsic ability of myeloid cells to elicit an inflammatory response when exposed to LPS may be impaired in the chronic absence of VIP.

Could a myeloid deficit in VIP KO mice contribute to their EAE resistance? The proinflammatory activities of autoreactive lymphocytes with Th1 and Th17 profiles have been shown to be essential for EAE development [36]. However, myeloid cells have been proposed as the ultimate effector cells that lead to the CNS damage [31]. Myeloid cells may contribute to the pathogenesis of EAE by secreting proinflammatory cytokines and chemokines, but they may also mediate tissue damage in this model by releasing enzymes that contribute to myelin destruction. For example, it has been shown that metalloproteases, such as MMP-2, released by macrophages cells in the CNS during EAE, enable the breakdown of the blood brain barrier and the infiltration of the CNS parenchyma by immune cells [37]. In addition to the resident microglia, abundant numbers of macrophages infiltrate the CNS during EAE. Depletion of macrophages with liposomal dichloromethylene diphosphonate (Cl_2_MDP) after EAE induction but before the appearance of clinical symptoms, led to the suppression of full EAE development [38]. Because this treatment eliminates bone marrow-derived macrophages leaving other populations such as microglia intact [39], the results highlight a crucial role for newly recruited macrophages for the development of the disease. Supporting the relevance of the proinflammatory role of macrophages in EAE, mice deficient in the macrophage chemokine CCL-1 (MCP-1) [40] and its receptor CCR-2 [41] are resistant to EAE, and genetic activation of the NF-κB pathway specifically in myeloid cells led to a more severe course of the disease [42]. We have found an impairment of NF-κB pathway signaling in VIP KO mice peritoneal cells, indicating an intrinsic defect in the response of these cells to at least some types of inflammatory stimuli. Several innate immune receptors activate the NF-κB pathway, including members of the IL-1R/TLR and TNF-R1 superfamilies. In addition, LPS directly activates NF-κB signaling through TLR4, which in turn triggers the expression of proinflammatory cytokines such as TNFα, IL-6 and IL-12. Although the specific mechanisms by which the NF-κB activation is impaired in VIP KO mice remain to be elucidated, this could be potentially due to abnormalities in TLR4 signaling in mice due to chronic absence of VIP.

At this point, we can only hypothesize why the lack of VIP results in decreased inflammation in the models of EAE and LPS-induced endotoxemia. At first glance, the data in this report contradicts the multiple existing studies demonstrating that VIP inhibits the release of proinflammatory mediators by murine macrophages [18], [21], [43], [44]. In these studies, VIP was added simultaneously or after the inflammatory stimulus. However, a few studies have shown that VIP can stimulate various aspects of the immune response under certain circumstances, for example when added to resting cells in culture. In this regard, VIP promoted the secretion of IL-6 by resting peritoneal macrophages *in vitro*, whereas it inhibited the production of this cytokine in the presence of LPS, suggesting that this peptide may exhibit dual actions depending on the activation status of the cell [45], [46]. In other organs, such as the anterior pituitary and bone marrow, VIP also stimulates IL-6 production, which in turn stimulates the release of pituitary hormones and the growth and differentiation of hematopoietic cells in the bone marrow [47]. Interestingly, Yadav *et al* have recently shown that VPAC1 KO mice present milder DSS-induced colitis compared to WT mice, suggesting that VIP may exert proinflammatory actions through this receptor [30]. Although these results suggest an impaired functionality of VIP KO of VPAC1 KO immune cells, other possibilities such as compensation by other factors such as peptides/hormones or their receptors may occur in mice when these molecules are chronically absent, and cannot be ruled out at this point. For example, we found a late increase in the systemic levels of IL-10 which could potentially contribute to the increased survival in the KO mice, although the importance of this mild induction needs to be further studied. Finally, an interesting finding was that despite the reduced mortality in VIP KO mice injected with LPS, we found a mild lung histological inflammation and higher levels of TNFα mRNA in this tissue in VIP KO mice in basal conditions. Szema *et al* previously reported similar results in VIP KO males, and agrees with the purported antiinflammatory actions of VIP [24]. It has been shown that prolonged administration of a low dose of LPS causes mild-degree inflammation which results in later poor responsiveness to LPS, a phenomenon known as “endotoxin tolerance” [48]. In would be interesting to determine if a continuous basal inflammation in VIP KO mice may be related to their decreased response to LPS. In conclusion, the unexpected decreased response of female VIP KO mice to LPS highlights the complexity of the actions of this peptide. This will need to be taken into account when therapies are designed to target this signaling pathway in inflammatory diseases.
