# Correction: VIP Deficient Mice Exhibit Resistance to Lipopolysaccharide Induced Endotoxemia with an Intrinsic Defect in Proinflammatory Cellular Responses

**DOI:** 10.1371/annotation/c70f1786-cb3f-4677-9a7c-0182dfbec74c

**Published:** 2013-06-12

**Authors:** Catalina Abad, Yossan-Var Tan, Gardenia Cheung-Lau, Hiroko Nobuta, James A. Waschek

The following sentence was incorrectly cited: 

"Interestingly, Yadav et al have recently shown that VPAC1 KO mice present milder DSS-induced colitis compared to WT mice, suggesting that VIP may exert proinflammatory actions through this receptor [30]."

The correct citation is [49], which is missing from the Reference list. Reference 49 is the following:

Yadav M, Huang MC, Goetzl EJ (2011) VPAC1 (vasoactive intestinal peptide (VIP) receptor type 1) G protein-coupled receptor mediation of VIP enhancement of murine experimental colitis. Cell Immunol. 267:124-32 

